# Brain *APOE* expression quantitative trait loci-based association study identified one susceptibility locus for Alzheimer’s disease by interacting with *APOE* ε4

**DOI:** 10.1038/s41598-018-26398-1

**Published:** 2018-05-23

**Authors:** Aiqian Zhang, Qingnan Zhao, Dabao Xu, Shan Jiang

**Affiliations:** 1 Department of Gynecology, Third Xiangya Hospital of Central South University, Changsha, Hunan China; 2 Department of Pediatrics, The University of Texas MD Anderson Cancer center, Houston, Texas USA; 3 Department of Psychiatry, Washington University School of Medicine, St. Louis, MO USA

## Abstract

Some studies have demonstrated interactions of AD-risk single nucleotide polymorphisms (SNPs) in non-*APOE* regions with *APOE* genotype. Nevertheless, no study reported interactions of expression quantitative trait locus (eQTL) for *APOE* with *APOE* genotype. In present study, we included 9286 unrelated AD patients and 8479 normal controls from 12 cohorts of NIA Genetics of Alzheimer’s Disease Data Storage Site (NIAGADS) and Alzheimer’s Disease Neuroimaging Initiative (ADNI). 34 unrelated brain eQTLs for *APOE* were compiled from BRAINEAC and GTEx. We used multi-covariate logistic regression analysis to identify eQTLs interacted with *APOE* ε4. Adjusted for age and gender, substantia nigra eQTL rs438811 for *APOE* showed significantly strong interaction with *APOE* ε4 status (OR, 1.448; CI, 1.124–1.430; *P*-value = 7.94 × 10^−6^). *APOE* ε4-based sub-group analyses revealed that carrying one minor allele T of rs438811 can increase the opportunity of developing to AD by 26.75% in *APOE* ε4 carriers but not in non-carriers. We revealed substantia nigra eQTL rs438811 for *APOE* can interact with *APOE* ε4 and confers risk in *APOE* ε4 carriers only.

## Introduction

Alzheimer’s Disease (AD) is the most common form of dementia with strong genetic etiology. Apolipoprotein E (*APOE*) ε4 allele has been universally confirmed as a strong risk factor for Late-Onset Alzheimer’s Disease (LOAD)^1,2^. APOE4, the isoform of APOE determined by ε4 allele, differs with other two isoforms APOE2 and APOE3 at protein structure, lipid association and receptor binding^3^. In most, if not all, putative AD pathogenic pathways, APOE4 either diminishes neuroprotection or augments neurotoxicity when compared with other two isoforms. These evidences which could explain the AD pathogenic nature of APOE4 include: APOE4 impairs synaptic repair and plasticity^4,5^; might be less efficient in transporting brain cholesterol^6,7^; increases Aβ aggregation and impairs clearance^8,9^; increases formation of neurofibrillary tangles^10^; decreases metabolic activity of neurons^11^.

Although APOE4 is neurotoxic remarkably and miscellaneously, not all *APOE* ε4 carriers developed to AD in a population, even for carriers of *APOE* ε4 homozygotes^12^. Likewise, not all *APOE* ε4 non-carriers are intact from AD. Except for *APOE* ε4, other genes were also identified to be associated with AD by genome-wide association studies (GWASs) in recent years^13–15^. Interestingly, Jun *et al*. revealed interaction of one of these susceptible genes *PICALM* with *APOE* ε4 on AD risk*—*genotypes at *PICALM* confer risk predominantly in *APOE* ε4 non-carriers^16^. In addition, *GAB2* and some other genes can also modify AD risk by interacting with *APOE* ε4^17–19^. Seemingly, these *APOE* ε4-interactive genes can give some reasons for the imperfect effect of *APOE* ε4, nevertheless, all of them are in non-*APOE* regions. Until now, no study has reported interactions of single nucleotide polymorphisms (SNPs) in *APOE* regulatory region with *APOE* genotypes, especially brain expression quantitative trait loci (eQTLs) for *APOE*. We speculate that some brain eQTLs can be related to AD by potentially regulating expression level of *APOE* ε4.

This study was aimed at identifying brain eQTLs for *APOE* which can interact with *APOE* ε4 allele to confer AD risk.

## Results

### Characteristics of included GWAS cohorts after related individual removal

Across the 13 GWAS cohorts, 1,320 AD patients and 1,502 healthy controls were identified by KING as duplicate samples or kin with a third degree (e.g. first cousin) or closer relationship. After excluding these samples, 9,286 unrelated AD patients and 8,479 healthy controls were retained.

Description of the 12 GWAS cohorts from NIA Genetics of Alzheimer’s Disease Data Storage Site (NIAGADS) and GWAS data from Alzheimer’s Disease Neuroimaging Initiative (ADNI) after related individual removal is shown in Table 1.

**Table 1:** Cohort description.

Abbreviated cohort name^*^	Ancestry	Cases/controls	Female (%)	Presence of *APOE* ε4 (%)
NIA-LOAD	mixed, 92.33% caucasian	993/884	62.30%	52%
ADC1	caucasian	1574/527	55.30%	57.90%
ADC2	caucasian	745/165	54.20%	55.10%
ADC3	caucasian	862/618	54.20%	40.70%
UPITT	mixed, 91.73% caucasian	1424/996	63.80%	42.20%
TGEN II	caucasian	1013/585	54.20%	48.50%
ROSMAP	caucasian	368/1326	69.10%	23.30%
WashU1	caucasian	403/225	58.10%	43.60%
MIRAGE	caucasian	603/885	59.70%	38.90%
ACT	unknown	567/1701	57.70%	26.10%
UMVUMSSM	unknown	1240/1230	62.50%	37.90%
MAYO	caucasian	841/1253	53.40%	42.70%
ADNI	mixed, 92.88% caucasian	213/347	47%	41.60%

^*^Cohort full names: NIA-LOAD, National Institute on Aging Genetics Initiative for Late-Onset Alzheimer’s Disease; ADC1, Alzheimer’s Disease Center Dataset 1; ADC2, Alzheimer’s Disease Center Dataset 2; ADC3, Alzheimer’s Disease Center Dataset 3; UPITT, University of Pittsburgh; TGEN II, Translational Genomics Research Institute II; ROSMAP, Religious Orders Study and Memory and Aging Project; WashU1, Washington University Dataset 1; MIRAGE, Multi Institutional Research on Alzheimer Genetics Epidemiology; ACT, Adult Changes in Thought; UMVUMSSM, University of Miami (UM), Vanderbilt University (VU) and Mount Sinai School of Medicine (MSSM); ADNI, Alzheimer’s Disease Neuroimaging Initiative.

### Brain tissue-specific eQTLs for APOE and determination of proxy SNPs

We collected 73 brain tissue-specific eQTLs from BRAINEAC (http://www.braineac.org/) and GTEx (https://www.gtexportal.org/home/). 34 out of the 73 eQTLs were determined by LDproxy (https://analysistools.nci.nih.gov/LDlink/?tab = ldproxy) as proxy eQTLs.

After excluding low-quality imputed eQTLs with imputation info score less than 0.9, all 34 proxy eQTLs were retained for further analysis. For the detailed information of the 34 proxy eQTLs, please refer to Table S1.

### rs438811 confers risk in APOE ε4 carriers predominantly

We used multivariate logistic regression analysis to identify eQTLs which can confer AD risk in the 34 proxy eQTLs for *APOE*. After adjustment for age and gender, substantia nigra eQTL rs438811 (odds ratio [OR], 2.343; 95% confidence interval [CI], 2.205–2.490; raw *P*-value = 7.49 × 10^−167^) was associated with AD (Table 2). After adjustment for age, gender and *APOE* ε4 status, rs438811 (OR, 1.049; 95% CI, 0.969–1.135; *P*-value = 0.237) was not associated with AD (Table 2). After introducing interaction item rs438811 genotype × *APOE* ε4 status into the model, rs438811 was found can confer AD risk by interacting with *APOE* ε4 status strongly (OR, 1.448; 95% CI, 1.231–1.704; *P*-value = 7.94 × 10^−6^; Table 3). Sub-group analysis showed rs438811 confer risk predominantly in *APOE* ε4 carriers (OR, 1.267; 95% CI, 1.124–1.430; *P*-value = 1.12 × 10^−4^; Table 3), which indicates carrying one minor allele T of rs438811 can increase the opportunity of developing to AD by 26.75% in *APOE* ε4 carriers. As shown in BRAINEAC, minor allele T of rs438811 was associated with increased *APOE* expression level in substantia nigra. The *APOE* eQTL *P*-values of rs438811 across the ten different brain regions were shown in Table S2.

**Table 2:** Substantia nigra eQTL rs438811 for *APOE* identified as a susceptibility locus for AD.

SNP	Minor allele	MAF^*^	Adjusted for age and gender		Adjusted for age, gender and *APOE* ε4 status	
			OR (95% CI)	*P*-value	OR (95% CI)	*P*-value
rs438811	T	0.287	2.343 (2.205–2.490)	7.49 × 10^−167^	1.049 (0.969–1.135)	0.237

^*^Weighed-average minor allele frequency.

**Table 3:** Interactive effect of substantia nigra eQTL rs438811 for *APOE* with *APOE* ε4 status on AD risk.

SNP	*APOE* ε4 carriers^*^		*APOE* ε4 non-carriers^*^		SNP × *APOE* ε4 status interaction^*^	
	OR (95% CI)	*P*-value	OR (95% CI)	*P*-value	OR (95% CI)	*P*-value
rs438811	1.267 (1.124–1.430)	1.12 × 10^−4^	0.882 (0.788–0.986)	0.028	1.448 (1.231–1.704)	7.94 × 10^−6^

^*^Adjusted for age and gender.

By querying Encyclopedia of DNA Elements (ENCODE), rs438811 was found to be target of transcription factors POLR2A and RPC155. rs483082, a brain eQTL for *APOE* which is in complete linkage with rs438811 and was also identified can interact with *APOE* ε4 status to confer AD risk in this study, was the target of multiple transcription factors: HNF4G, CEBPB, MXI1, HDAC2, SP1, RFX5, MAX, EP300, JUND, FOSL2, ZBTB7A and CEBPD. rs483082 was also found be to located in a DNase I hypersensitivity cluster.

## Discussion

*APOE* has long been a widely-investigated gene since the identification of its association with AD. Many studies have reported the relations between *APOE* genotypes and AD-related traits, such as cerebral spinal fluid (CSF) biomarkers^20–22^, brain morphology changes^23–25^, and particular cognitive measures^23,26,27^. However, except for *APOE* ε4, no locus encompassing the *APOE* region, including brain eQTLs for *APOE*, were identified as a conferring risk for AD or AD-related traits. This study identified AD risk-associated brain eQTL for *APOE* by incorporating multiple GWAS cohorts.

The susceptibility locus rs438811 identified is in complete linkage with another brain eQTL for *APOE*–rs483082–which was associated with AD in summary statistics of International Genomics of Alzheimer’s project (IGAP) and was reported to associate with AD in a Japanese population^15,28^. rs483082 was also associated with AD and confer AD risk in *APOE* ε4 carriers only in this study (data not shown). Furthermore, rs483082 was also reported to associate with lipid level^29^. Abnormal lipid metabolism has long been demonstrated to as being involved in AD pathology^30–32^. Our results revealed that the eQTL may influence the progression of AD *APOE* ε4 carriers by increasing the expression level of *APOE*. -491A/T, or rs449647, a polymorphism located in *APOE* transcriptional regulatory region, is the earliestly-reported *APOE* expression-associated variant by luciferase/β-galactosidase activity assay related to AD independent of *APOE* ε4 dosage^33^. In contrast to the earliest AD-associated *APOE* eQTL, the *APOE* eQTL identified in this study affect *APOE* expression in a brain region-specific manner. We also analyzed the association of -491A/T with AD (Table S3) and its interactive effect with *APOE* ε4 status on AD risk (Table S4). In consistent with the result from the study applied luciferase/β-galactosidase activity assay^33^, −491A/T confer AD risk independent of *APOE* ε4 status. rs438811 is a substantia nigra-specific *APOE* eQTL. As a brain substructure dysfunction of which contributes to extrapyramidal signs (EPS), pathological changes of substantia nigra are responsible for EPS and aggravated EPS in AD patients^34^. As to how the brain *APOE* eQTL influence the progression of AD, one most probable explanation is the neurotoxic effect of increased APOE4 expression level in substantia nigra. Nevertheless, pathological mechanism of the brain *APOE* eQTL still needs to be unveiled by molecular biological experiments.

In summary, this is a pilot study associating brain *APOE* eQTLs to AD risk. It identified a novel SNPs associated with AD by interacting with *APOE* ε4 status and potentially regulating expression level of *APOE*.

## Methods

### Compiling of brain APOE eQTLs

Brain *APOE* eQTLs studied in this multi-cohort gene-wide association study were collected by querying BRAINEAC (http://www.braineac.org/) and GTEx (https://www.gtexportal.org/home/) databases. BRAINEAC provides the gene expression across ten brain tissues (cerebellar cortex, frontal cortex, hippocampus, medulla, occipital cortex, putamen, substantia nigra, thalamus, temporal cortex and intralobular white matter) from 134 healthy control individuals. GTEx provides the gene expression across thirteen brain tissues (amygdala, anterior cingulate cortex, caudate, cerebellar hemisphere, cerebellum, cortex, frontal cortex, hippocampus, hypothalamus, nucleus accumbens, putamen, spinal cord and substantia nigra) with the sample sizes ranged from 80 to 154. The cis brain eQTLs with *P*-values less than 1 × 10^−3^ and located within up- or down-stream 10 Mb boundary of the *APOE* gene were retrieved from the two eQTL databases. To reduce redundant computation for the AD association analysis, brain eQTLs within the same linkage disequilibrium (LD) block were pruned and one eQTL was chosen to serve as the proxy for the LD block. Proxy brain eQTLs were determined by LDproxy (https://analysistools.nci.nih.gov/LDlink/?tab=ldproxy) with a threshold of LD r^2^ ≥ 0.8.

### Subjects

In this study, we included a total of 13 AD GWAS cohorts. 12 out of them were from NIAGADS. The criteria for inclusion of cohorts from NIAGADS was carrying covariate information on age, gender and *APOE* genotype. For detailed information on the 12 cohorts from NIAGADS, please refer to Table S5. The last cohort was from ADNI. GWAS data from ADNI were generated as previously described and obtained from the ADNI database (http://www.loni.ucla.edu/ADNI/)^35^. Finally, a total of 10606 AD patients and 9981 healthy controls were included in this study.

### Identification and exclusion of related individuals

We used KING to identify and exclude duplicate samples and kin with a third degree (e.g. first cousin) or closer relationship within and across datasets^36^.

### Quality control of chromosome 19

For the purpose of imputation, we extracted all SNPs in chromosome 19, where *APOE* is located, from each GWAS dataset. Standard quality control was then applied using Plink 1.9^37^. For each dataset, SNPs with a call rate of less than 99%, minor allele frequency of less than 1% and violation of Hardy-Weinberg equilibrium in controls (*P* < 1 × 10^−4^) were removed. Samples with a call rate of less than 90% were removed.

### Imputation of chromosome 19

Because all the brain eQTLs collected in this study are cis eQTLs which are located within up- or down-stream 10 Mb boundary of the *APOE* gene, only chromosome 19 was imputed to reduce computation burden. The SNPs of chromosome 19 were prephased using SHAPEIT2 for each dataset^38^. SNPs were then imputed to a reference panel of 1000 Genome Project Phase 3 by IMPUTE2^39^. An imputation info score cutoff of 0.9 was applied to exclude low-quality imputed SNPs. After imputation, collected eQTLs from the two databases for brain tissues were extracted.

### Identification of population substructure

To adjust for confounding effect of population substructure in our data, we calculated eigenvectors of individuals through whole genome-wide principal component analysis (PCA) before chromosome 19 SNPs were extracted using Plink 1.9^37^. All Principal components (PCs, from PC1 to PC20) were used for confounding adjustment.

### Combination of genotyped and imputed data

In this study, the genotyped and imputed brain eQTLs for *APOE* were inconsistent among different datasets. In order to combine all the GWAS datasets for AD association analysis, imputed high-quality brain eQTL with high confidence (genotype probability greater than 0.8) were converted to be simulated genotype data by fcGENE for each individual^40^. Simulated genotype data were then combined with originally genotyped data.

### Statistical analysis

After adjusting for age, gender and all PCs, these proxy brain eQTLs were tested for associations with AD with or without adjustment for *APOE* ε4 status through multivariate logistic regression analysis. eQTLs were coded as 0, 1, or 2 according to their number of minor alleles. *APOE* ε4 status was coded as 0 or 1 according to absence (*APOE* ε2/2, ε2/3 and ε3/3 subjects) or presence (*APOE* ε2/4, ε3/4 and ε4/4 subjects) of *APOE* ε4. Interaction item SNP genotype × *APOE* ε4 status was then introduced into the model to investigate the interactive effect of SNP and *APOE* ε4 status on AD risk. To account for multiple testing, we used the Bonferroni correction and considered significant only those brain eQTLs for which *P*-value < 0.05/34 = 0.05/34 = 1.47 × 10^−3^. All statistical calculations were performed using R^41^.

### Biological function annotation of identified *APOE* ε4 status-interactive brain eQTLs for *APOE*

Biological function annotation of identified *APOE* ε4 status-interactive brain eQTLs for *APOE* was performed via querying Encyclopedia of DNA Elements (ENCODE, https://www.encodeproject.org/).

### Data availability statement

The brain eQTL data for *APOE* can be retrieved from Braineac (http://www.braineac.org/) and GTEx (https://www.gtexportal.org/home/). The GWAS datasets used in this study can be applied and downloaded from NIAGADS (https://www.niagads.org/) and ADNI (http://adni.loni.usc.edu/).

## Electronic supplementary material

Supplementary Information
