# Supplementary material for: Brain APOE expression quantitative trait loci-based association study identified one susceptibility locus for Alzheimer’s disease by interacting with APOE ε4
Source: Sci Rep. 2018 May 23;8:8068. doi: 10.1038/s41598-018-26398-1 (PMC5966425; doi:10.1038/s41598-018-26398-1)
Supplement: Supplementary file 1 — Supplementary Information [file 41598_2018_26398_MOESM1_ESM.docx]

**Brain *APOE* expression quantitative trait loci-based association study identified one susceptibility locus for Alzheimer's disease by interacting with *APOE* ε4**

**Aiqian Zhang^1^, Qingnan Zhao^2^, Dabao Xu^1,*^, Shan Jiang^3,*^**

^1^Department of Gynecology, Third Xiangya Hospital of Central South University, Changsha, Hunan, China

^2^Department of Pediatrics, The University of Texas MD Anderson Cancer center, Houston, Texas, USA

^3^Department of Psychiatry, Washington University School of Medicine, St. Louis, MO, USA

***Corresponding authors:**

1- Dabao Xu, MD

Email: [dabaoxu@yahoo.com](mailto:dabaoxu@yahoo.com)

Department of Gynecology, Third Xiangya Hospital of Central South University, 138 Tongzipo Rd, Changsha, Hunan 410013, China.

2- Shan Jiang, PhD

Email: [shannjiang@hotmail.com](mailto:shannjiang@hotmail.com)

Department of Psychiatry, Washington University School of Medicine, St. Louis, MO, USA.

Supplementary information

**Table S1.** Complete list of the 34 proxy brain eQTLs for *APOE*

**Table S2.** *APOE* eQTL *P*-values of rs438811 for the ten different brain regions

**Table S3.** -491A/T was associated with AD

**Table S4.** -491A/T was associated with AD independent of APOE ε4 status

**Table S5.** Detailed information of the 14 high-throughput genotyping cohorts from NIA Genetics of Alzheimer's Disease Data Storage Site (NIAGADS)

**Table S1. Complete list of the 34 proxy brain eQTLs for *APOE***

| **SNP** | **Chr.** | **Position*** | **Position relative to *APOE*** | **LD with rs429358 (R^2^)†** | **LD with rs7412 (R^2^)†** | ***P*-value for eQTL** | **Normalized effect size** | **Brain tissue** | **Source** | **P-value for AD association‡** | **P-value for AD association in *APOE* ε4 carriers§** | **P-value for AD association in *APOE* ε4 non-carriers§** |
| --- | --- | --- | --- | --- | --- | --- | --- | --- | --- | --- | --- | --- |
| rs55848260 | chr 19 | 45080369 | Upstream 328670 bases | 0.003 | 0.001 | 5.20E-04 | Not available | Cerebellar cortex | BRAINEAC | 3.13E-01 | 5.34E-02 | 7.29E-01 |
| rs74439218 | chr 19 | 45196254 | Upstream 212785 bases | 0.004 | 0.001 | 4.40E-04 | Not available | Cerebellar cortex | BRAINEAC | 9.32E-01 | 5.96E-01 | 7.44E-01 |
| rs34342452 | chr 19 | 46000901 | Downstream 588251 bases | 0.002 | 0 | 4.80E-04 | Not available | Cerebellar cortex | BRAINEAC | 4.17E-01 | 5.60E-01 | 5.49E-01 |
| rs2722667 | chr 19 | 44912270 | Upstream 496769 bases | 0.002 | 0.002 | 6.50E-04 | Not available | Cerebellar cortex | BRAINEAC | 5.58E-01 | 6.56E-01 | 6.71E-01 |
| rs35864669 | chr 19 | 45591771 | Downstream 179121 bases | 0 | 0.001 | 1.20E-04 | Not available | Hippocampus | BRAINEAC | 2.44E-01 | 9.27E-02 | 9.71E-01 |
| rs28715089 | chr 19 | 46151134 | Downstream 738484 bases | 0.001 | 0 | 2.60E-04 | Not available | Hippocampus | BRAINEAC | 7.67E-01 | 4.51E-01 | 7.27E-01 |
| rs404982 | chr 19 | 44960218 | Upstream 448821 bases | 0.003 | 0.001 | 5.30E-04 | Not available | Hippocampus | BRAINEAC | 9.12E-01 | 5.77E-01 | 7.95E-01 |
| rs59135122 | chr 19 | 45120905 | Upstream 288134 bases | 0 | 0 | 1.20E-04 | Not available | Medulla | BRAINEAC | 5.70E-01 | 6.28E-01 | 7.86E-01 |
| rs1967311 | chr 19 | 45819307 | Downstream 406657 bases | 0.004 | 0 | 5.90E-04 | Not available | Medulla | BRAINEAC | 6.41E-01 | 4.73E-01 | 1.97E-01 |
| rs7252108 | chr 19 | 44619542 | Upstream 789497 bases | 0 | 0.001 | 4.00E-04 | Not available | Occipital cortex | BRAINEAC | 1.26E-01 | 5.37E-01 | 1.70E-01 |
| rs12975617 | chr 19 | 46208023 | Downstream 795373 bases | 0.001 | 0.001 | 7.30E-05 | Not available | Occipital cortex | BRAINEAC | 9.33E-02 | 1.58E-02 | 7.39E-01 |
| rs12972749 | chr 19 | 44898017 | Upstream 511022 bases | 0.003 | 0.001 | 5.90E-04 | Not available | Occipital cortex | BRAINEAC | 4.26E-01 | 3.72E-01 | 7.77E-01 |
| rs12462616 | chr 19 | 46310512 | Downstream 897862 bases | 0.001 | 0 | 2.40E-04 | Not available | Occipital cortex | BRAINEAC | 2.00E-01 | 9.04E-02 | 7.16E-01 |
| rs846881 | chr 19 | 45078553 | Upstream 330486 bases | 0.003 | 0 | 2.70E-04 | Not available | Putamen | BRAINEAC | 5.55E-01 | 2.54E-01 | 8.46E-01 |
| rs112401916 | chr 19 | 45645553 | Downstream 232903 bases | 0.003 | 0 | 1.30E-04 | Not available | Putamen | BRAINEAC | 4.06E-03 | 4.20E-02 | 2.71E-02 |
| rs117560401 | chr 19 | 45125369 | Upstream 283670 bases | 0 | 0.001 | 1.40E-04 | Not available | Putamen | BRAINEAC | 6.05E-01 | 9.93E-01 | 5.89E-01 |
| rs438811 | chr 19 | 45416741 | Downstream 4091 bases | 0.404 | 0.202 | 3.10E-04 | Not available | Substantia nigra | BRAINEAC | 2.37E-01 | 1.12E-04 | 2.78E-02 |
| rs73035960 | chr 19 | 44514195 | Upstream 894844 bases | 0 | 0 | 9.40E-05 | Not available | Substantia nigra | BRAINEAC | 9.65E-01 | 9.13E-01 | 8.76E-01 |
| rs2972557 | chr 19 | 45360573 | Upstream 48466 bases | 0.001 | 0 | 9.70E-04 | Not available | Temporal cortex | BRAINEAC | 6.05E-01 | 6.59E-01 | 8.03E-01 |
| rs2103262 | chr 19 | 45029894 | Upstream 379145 bases | 0.004 | 0.001 | 3.60E-04 | Not available | Temporal cortex | BRAINEAC | 7.29E-01 | 6.73E-01 | 3.34E-01 |
| rs3916855 | chr 19 | 45860202 | Downstream 447552 bases | 0 | 0 | 5.80E-04 | Not available | Thalamus | BRAINEAC | 2.64E-01 | 7.21E-01 | 1.10E-01 |
| rs62109650 | chr 19 | 46052898 | Downstream 640248 bases | 0 | 0 | 4.70E-04 | Not available | Intralobular white matter | BRAINEAC | 1.09E-01 | 2.01E-03 | 6.80E-01 |
| rs74253343 | chr 19 | 45670248 | Downstream 257598 bases | 0.002 | 0 | 1.70E-04 | Not available | Intralobular white matter | BRAINEAC | 5.63E-01 | 3.57E-01 | 9.94E-01 |
| rs2030404 | chr 19 | 44043448 | Upstream 1365591 bases | 0.001 | 0 | 9.59E-04 | Not available | Cerebellum | BRAINEAC | 2.44E-02 | 1.19E-03 | 7.87E-01 |
| rs2682592 | chr 19 | 43940597 | Upstream 1468442 bases | 0 | 0.002 | 6.46E-04 | Not available | Cerebellum | BRAINEAC | 9.88E-01 | 3.82E-01 | 5.07E-01 |
| rs17206386 | chr 19 | 36931957 | Upstream 8477082 bases | 0 | 0 | 6.62E-04 | Not available | Cerebellum | BRAINEAC | 2.92E-01 | 3.07E-01 | 5.18E-01 |
| rs2972448 | chr 19 | 38168913 | Upstream 7240126 bases | 0.001 | 0 | 3.97E-04 | Not available | Cerebellum | BRAINEAC | 6.51E-01 | 6.83E-01 | 8.87E-01 |
| rs4803551 | chr 19 | 38399461 | Upstream 7009578 bases | 0 | 0.001 | 4.47E-04 | Not available | Cerebellum | BRAINEAC | 5.65E-01 | 7.89E-01 | 6.01E-01 |
| rs4802074 | chr 19 | 40848081 | Upstream 4560958 bases | 0 | 0 | 7.77E-04 | Not available | Cerebellum | BRAINEAC | 4.28E-02 | 4.60E-01 | 6.09E-02 |
| rs4490099 | chr 19 | 40731491 | Upstream 4677548 bases | 0 | 0 | 9.66E-04 | Not available | Cerebellum | BRAINEAC | 2.25E-02 | 8.36E-01 | 6.81E-03 |
| rs1628394 | chr 19 | 38535008 | Upstream 6874031 bases | 0.003 | 0 | 9.54E-04 | Not available | Cerebellum | BRAINEAC | 2.45E-03 | 1.63E-01 | 6.32E-03 |
| rs2909088 | chr 19 | 38176548 | Upstream 7232491 bases | 0.001 | 0 | 3.97E-04 | Not available | Cerebellum | BRAINEAC | 6.90E-01 | 6.98E-01 | 9.29E-01 |
| rs147910350 | chr 19 | 46146762 | Downstream 734112 bases | 0 | 0 | 2.90E-06 | -2 | Frontal cortex (BA9) and Nucleus accumbens (basal ganglia) | BRAINEAC | 6.39E-01 | 9.71E-01 | 5.97E-01 |
| rs35899189 | chr 19 | 45025134 | Upstream 383905 bases | 0.005 | 0.001 | 6.60E-06 | -0.25 | Brain - Nucleus accumbens (basal ganglia) | BRAINEAC | 8.81E-01 | 8.41E-01 | 8.07E-01 |

*Build 37, assembly hg19.

†Information of linkage disequilibrium with the two *APOE* genotype determinant SNPs was retrieved from LDlink: <https://analysistools.nci.nih.gov/LDlink/>

‡Adjusted for age, gender and *APOE* ε4 status

§Adjusted for age and gender

**Table S2. *APOE* eQTL *P*-values of rs438811 for the ten different brain regions**

| **Brain region** | **P-value** |
| --- | --- |
| Cerebellar cortex | 5.90E-01 |
| Frontal cortex | 3.30E-01 |
| Hippocampus | 8.30E-02 |
| Medulla | 6.70E-01 |
| Occipital cortex | 6.80E-01 |
| Putamen | 6.20E-01 |
| Substantia nigra | 3.10E-04 |
| Temporal cortex | 9.30E-01 |
| Thalamus | 2.20E-01 |
| Intralobular white matter | 4.60E-01 |
| average all | 3.60E-01 |

**Table S3. -491A/T was associated with AD**

|  |  |  | Adjusted for age and gender | | Adjusted for age, gender and *APOE* ε4 status | |
| --- | --- | --- | --- | --- | --- | --- |
| SNP | Minor allele | MAF* | OR (95% CI) | *P*-value | OR (95% CI) | *P*-value |
| -491A/T | T | 0.127 | 0.631 (0.576-0.692) | 7.30 × 10^-23^ | 0.801 (0.726-0.885) | 1.17 × 10^-5^ |

*Weighed-average minor allele frequency.

**Table S4. -491A/T was associated with AD independent of APOE ε4 status**

|  | *APOE* ε4 carriers* | | *APOE* ε4 non-carriers* | | SNP × *APOE* ε4 status interaction* | |
| --- | --- | --- | --- | --- | --- | --- |
| SNP | OR (95% CI) | *P*-value | OR (95% CI) | *P*-value | OR (95% CI) | *P*-value |
| -491A/T | 0.785 (0.663-0.931) | 5.14 × 10^-3^ | 0.815 (0.721-0.921) | 1.05 × 10^-3^ | 1.639 (0.008-328.080) | 0.855 |

*Adjusted for age and gender.

**Table S5. Detailed information of the 14 high-throughput genotyping cohorts from NIA Genetics of Alzheimer's Disease Data Storage Site (NIAGADS)**

| **Abbreviated cohort name*** | **NIAGADS cohort accession number** | **Weblink for description** |
| --- | --- | --- |
| NIA-LOAD | NG00020 | <https://www.niagads.org/datasets/ng00020> |
| ADC1 | NG00022 | <https://www.niagads.org/datasets/ng00022> |
| ADC2 | NG00023 | <https://www.niagads.org/datasets/ng00023> |
| ADC3 | NG00024 | <https://www.niagads.org/datasets/ng00024> |
| UPITT | NG00026 | <https://www.niagads.org/datasets/ng00026> |
| TGEN II | NG00028 | <https://www.niagads.org/datasets/ng00028> |
| ROSMAP | NG00029 | <https://www.niagads.org/datasets/ng00029> |
| WashU1 | NG00030 | <https://www.niagads.org/datasets/ng00030> |
| MIRAGE | NG00031 | <https://www.niagads.org/datasets/ng00031> |
| ACT | NG00034 | <https://www.niagads.org/datasets/ng00034> |
| UMVUMSSM | NG00042 | <https://www.niagads.org/datasets/ng00042> |
| MAYO | NG00043 | <https://www.niagads.org/datasets/ng00043> |

*Cohort full names: NIA-LOAD, National Institute on Aging Genetics Initiative for Late-Onset Alzheimer's Disease; ADC1, Alzheimer's Disease Center Dataset 1; ADC2, Alzheimer's Disease Center Dataset 2; ADC3, Alzheimer's Disease Center Dataset 3; UPITT, University of Pittsburgh; TGEN II, Translational Genomics Research Institute II; ROSMAP, Religious Orders Study and Memory and Aging Project; WashU1, Washington University Dataset 1; MIRAGE, Multi Institutional Research on Alzheimer Genetics Epidemiology; ACT, Adult Changes in Thought; UMVUMSSM, University of Miami (UM), Vanderbilt University (VU) and Mount Sinai School of Medicine (MSSM); ADNI, Alzheimer's Disease Neuroimaging Initiative.
